# Supplementary material for: The Relationship Between Widespread Pollution Exposure and Oxidized Products of Nucleic Acids in Seminal Plasma and Urine in Males Attending a Fertility Center
Source: Int J Environ Res Public Health. 2020 Mar 13;17(6):1880. doi: 10.3390/ijerph17061880 (PMC7143937; doi:10.3390/ijerph17061880)
Supplement: Supplementary file 1 [file ijerph-17-01880-s001.pdf]

International Journal of Environmental Research and Public Health

The Relationship Between Widespread Pollution Exposure and Oxidized Products of Nucleic acids in Seminal Plasma and Urine in Males Attending a Fertility Center

Supplementary Material

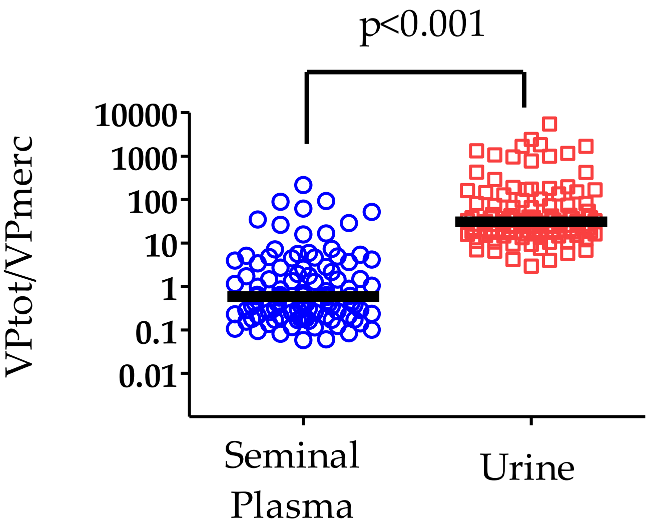

Figure S1. Vinylphenol glucuro- and sulpho-conjugates (VP tot) and Vinylphenol mercapturic acid (VP merc) ratio in SP and urine.

Table S1. Pearson's correlation coefficients of biomarkers in SP *vs* urine.

Seminal Plasma *vs* Urine	R
DHBMA	0.49 **
DHPMA	0.23 *
SPMA	N.S.
SBMA	0.23 *
VPmerc	0.54 **
VPtot	N.S.
NOHtot	NS
Cot	0.94 **

R* = *p* <0.05; R** = *p* < 0.01**.

Table S2. SP: Pearson's correlation coefficients between biomarkers of exposure.

Seminal Plasma	DHBMA	DHPMA	SPMA	SBMA	VPMerc	VP_tot	NOHtot
DHBMA	1						
DHPMA	0.845**	1					
SPMA	0.630**	0.471**	1				
SBMA	0.697**	0.735**	0.480**	1			
VPmerc	0.589**	0.333**	0.678**	0.426**	1		
VPtot	0.277*	0.391**	N.S.	0.373**	N.S.	1	
NOHtot	N.S.	N.S.	N.S.	0.396**	N.S.	0.597**	1

R* = p< 0.05; R** = p< 0.01; N.S. = non-significant.

Table S3. Urine: Pearson's correlation coefficients between biomarkers of exposure.

Urine	DHBMA	DHPMA	SPMA	SBMA	VPMerc	VP_tot	NOHtot	t,t-MA	MA	PGA	AFIEM
DHBMA	1										
DHPMA	0.821**	1									
SPMA	0.536**	0.417**	1								
SBMA	0.300**	N.S.	0.259*	1							
VPmerc	0.716**	0.684**	0.255*	N.S.	1						
VPtot	N.S.	N.S.	0.223*	N.S.	N.S.	1					
NOHtot	0.571**	0.486**	0.467**	0.279*	0.402**	0.491**	1				
t,t-MA	0.445**	0.423**	0.439**	0.300**	N.S.	0.245*	0.444**	1			
MA	0.702**	0.593**	0.558**	0.367**	0.295**	0.519**	0.624**	0.575**	1		
PGA	0.279**	0.320**	0.256**	0.444**	N.S.	0.254**	0.264**	0.517**	0.465**	1	
AFIEM	0.338**	0.371**	0.326**	0.425**	N.S.	0.243*	0.260**	0.344**	0.489**	0.452**	1

R* = p <0.05; R** = p <0.01; N.S. = non-significant.
